# Identification of KRAS mutation in rectal cancer based on a 2.5D deep learning model

**DOI:** 10.3389/fonc.2026.1763859

**Published:** 2026-02-25

**Authors:** Chengmeng Zhang, Jinge Li, Peng Chen, Yanyan Zhou, Jian Shen, Guanfeng Chen

**Affiliations:** 1 Radiology Department of Huzhou Central Hospital, Fifth School of Clinical Medicine of Zhejiang Chinese Medical University, Huzhou, China; 2 Medical School of Huzhou University, Huzhou, China; 3 Radiology Department of Changxing County Traditional Chinese Medicine Hospital, Huzhou, China; 4 Radiology Department of Quanzhou First Hospital Affiliated to Fujian Medical University, Quanzhou, China

**Keywords:** deep transfer learning, gene mutation, radiomics, rectal cancer, X-ray computed tomography

## Abstract

**Objective:**

To explore the utility of a 2.5D deep transfer learning (DTL) model for distinguishing between Kirsten rat sarcoma viral oncogene (KRAS) mutant and wild-type phenotypes in patients with rectal cancer (RC).

**Methods:**

We retrospectively analyzed 138 patients with pathologically confirmed RC who underwent next-generation sequencing to detect KRAS mutations. Among these, 43 KRAS mutant and 95 wild-type cases were enrolled and divided randomly into a training set (30 mutant, 66 wild-type) and a validation set (13 mutant, 29 wild-type) in a 7:3 ratio. Tumor regions of interest (ROIs) were delineated manually slice-by-slice in thin-section arterial-phase computed tomography images. DTL and radiomic features were extracted from ROIs using 2.5D deep learning and traditional radiomic approaches, respectively. After feature-dimensionality reduction and selection, six machine learning models were employed to construct radiomic models and 2.5D deep learning models. The diagnostic performance of each model was evaluated using the area under the receiver operating characteristic curve (AUC).

**Results:**

After feature selection, 10 radiomic features and 17 DTL features were included for model construction. The AUCs for the radiomic models ranged from 0.808–0.988 in the training set and 0.521–0.672 in the validation set, with the XGBoost classifier achieving the optimal performance (AUC = 0.672) in the validation set. The AUCs for the 2.5D deep learning models ranged from 0.950–1.000 in the training set and 0.788–0.913 in the validation set, with the support vector machine classifier demonstrating the best diagnostic efficacy (AUC = 0.913) in the validation set.

**Conclusion:**

A 2.5D deep learning model can effectively distinguish between KRAS mutant and KRAS wild-type RC, outperforming traditional radiomic models. It provides a novel non-invasive approach for the preoperative assessment of KRAS mutation status.

## 1 Introduction

Rectal cancer (RC) is a malignant gastrointestinal tumor with high morbidity and mortality, posing a significant threat to human health (1). Numerous previous studies have established the significance of the KRAS (Kirsten rat sarcoma viral oncogene homolog) gene and demonstrated its close correlation with the prognosis of RC (2). Several studies have also identified KRAS mutation as a negative predictive biomarker for treatment with epidermal growth factor receptor antibodies (3).Traditionally, KRAS mutations are detected using surgical or needle biopsies; however, these are invasive, carry certain risks, and may not be tolerated by some patients with advanced disease (4). Exploring non-invasive and efficient methods for KRAS mutation detection has thus become a focus of clinical research.

Computed tomography (CT), as a routine preoperative imaging modality for RC, can clearly display tumor morphology, density, and enhancement patterns, providing abundant biological information. In addition, artificial intelligence, which quantifies tumor characteristics by analyzing quantitative features imperceptible to the human eye, has shown promising potential for tumor classification, prognosis evaluation, and in other fields (5, 6). Previous studies have suggested associations between KRAS mutation and factors such as tumor N stage and maximum tumor diameter (7). Additionally, high D* and low D values derived from intravoxel incoherent motion diffusion-weighted imaging have been reported to correlate significantly with KRAS mutation (8). However, these findings lack consensus, with substantial variations across studies, making it difficult to establish unified standards.

Deep learning, particularly convolutional neural networks, have recently achieved notable progress in terms of disease diagnosis, prognosis prediction, and treatment response assessment (9). Lui et al. (10) utilized the MobileNetV2 deep learning network to extract high-dimensional image features, achieving favorable performance in predicting KRAS mutation status in patients with RC. Similar to conventional radiomic methods, however, this method required manual delineation of the three-dimensional (3D) tumor region, resulting in substantial workload and time costs. The present study accordingly aimed to construct a 2.5D deep learning-based predictive model for distinguishing between KRAS mutant and wild-type RC. We also aimed to compare the diagnostic performance of this model with conventional radiomic models to explore the clinical application value of this 2.5D deep learning model for the preoperative, non-invasive assessment of KRAS mutation status.

## 2 Materials and methods

### 2.1 Data collection

This retrospective study analyzed data for patients with pathologically confirmed RC after surgery, and who underwent KRAS mutation detection via next-generation sequencing at Quanzhou First Affiliated Hospital of Fujian Medical University between April 2022 and February 2025. All patients underwent whole-abdominal plain CT plus dual-phase enhanced scanning within 2 weeks before surgery.

The inclusion criteria were pathologically confirmed RC with gene detection results, complete imaging data with excellent image quality, and patients with intestinal diseases such as ulcerative colitis or Crohn’s disease. The exclusion criteria were receipt of anti-tumor treatments (e.g., radiotherapy, chemotherapy) prior to gene detection, tumor involvement extending to the sigmoid colon or descending colon, tumor invasion of surrounding tissues leading to unfeasible region of interest (ROI) delineation, and ROI that failed to fully cover the entire tumor tissue.

After applying the inclusion and exclusion criteria, a total of 138 patients were enrolled in the study (93 men, 45 women; age 33–87 years), including 43 KRAS mutant and 95 wild-type cases. This retrospective study was approved by the Ethics Committee of Quanzhou First Affiliated Hospital of Fujian Medical University (Approval No.: Quan-Yi-Lun-2024-K133), in compliance with the Declaration of Helsinki. The need for informed consent was waived for all patients.

### 2.2 Equipment and scanning protocol

Routine whole-abdominal scanning was carried out using a GE 64-slice CT scanner (Optima CT660, General Electric, USA). Patients were placed in a supine position, with a scanning range from the dome of the diaphragm to the lower edge of the pubic symphysis. The scanning parameters were as follows: tube voltage 120 kV, automatic tube current, slice thickness 5 mm, and slice interval 5 mm. Enhanced scanning was carried out using the time method: after plain scanning, iohexol (iodine concentration: 300 mg/mL) was injected via an antecubital vein cannula using a high-pressure injector at a flow rate of 3.0 mL/s. Arterial- and venous-phase scans were initiated at 30 s and 60 s after the start of injection, respectively.

### 2.3 ROI delineation

**Figure 1:**
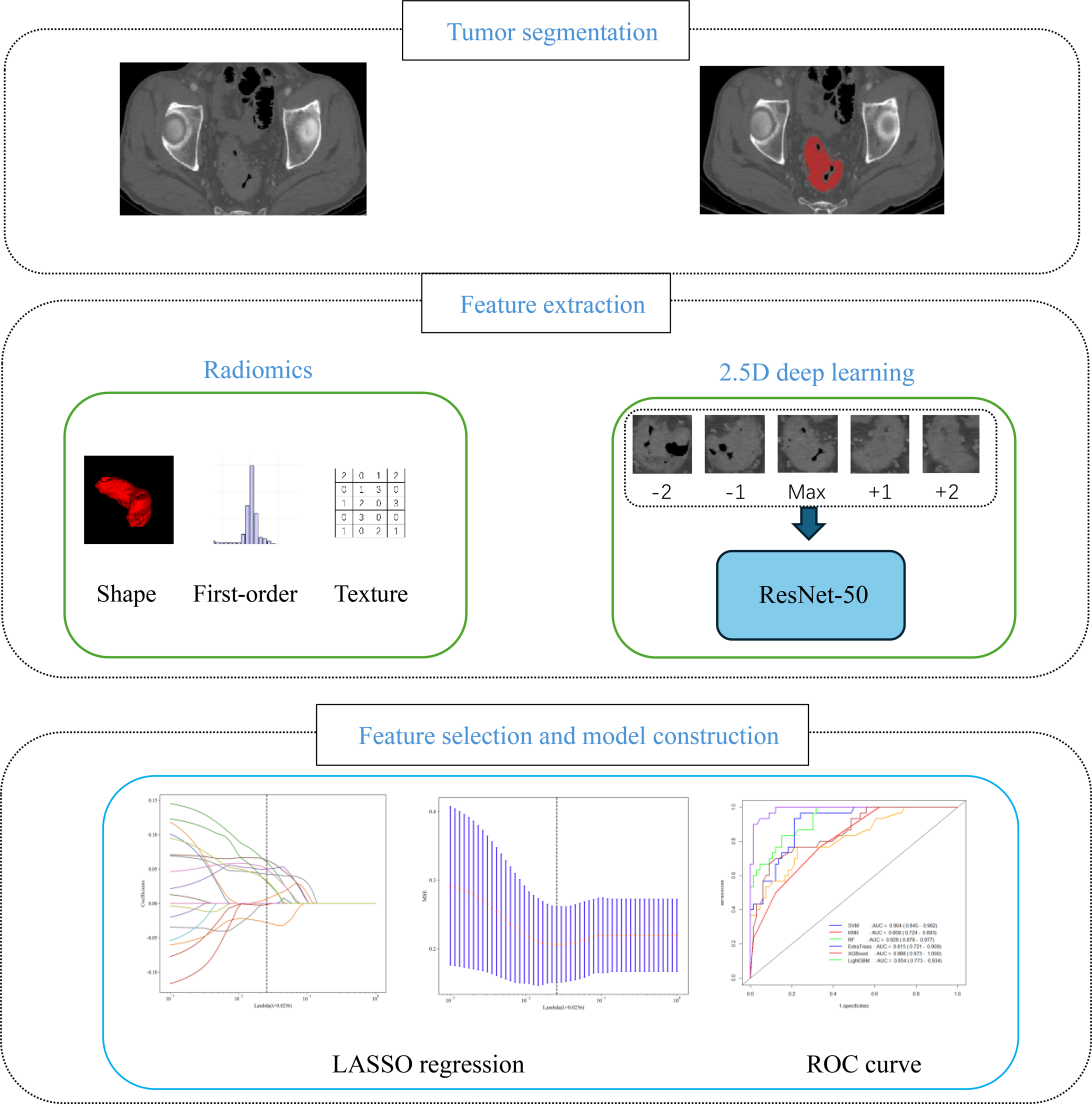
Workflow of the key steps in our study.

All arterial-phase CT images were standardized to a voxel spacing of 1 mm × 1 mm × 1 mm to ensure a consistent resolution. ROIs were delineated by two radiologists (Physician A with > 5 years of diagnostic experience and Physician B with > 15 years of diagnostic experience) using ITK-SNAP software (version 4.2.2). The transverse tumor range was delineated slice-by-slice as the ROI to extract radiomic and 2.5D deep transfer learning (DTL) features (**Figure 1**).

### 2.4 Extraction of DTL and radiomic features

Radiomic features were extracted from segmented images using the open-source Python library PyRadiomics tool (version 3.0.1). Scikit-Learn (version 1.0.2) was employed to implement machine learning models for classification, regression, and feature selection. Deep learning model development was carried out using PyTorch (version 1.11.0), as a widely adopted deep learning framework. To improve computational efficiency, CUDA (version 11.3.1) and CUDNN (version 8.2.1) were used to reduce the training time and enable more efficient model optimization. Unlike traditional 2D deep learning models that only use the maximum cross-section for training, the 2.5D deep learning approach selected five slices for each lesion: the maximum slice and the adjacent slices at the ±1 and ±2 positions. This method retains spatial information to a certain extent and ensures sufficient capture of image structural details. The ResNet50 deep convolutional network architecture was subsequently selected as the basic DTL model in the PyTorch deep learning library. Image features were extracted using convolutional layer modules, and DTL features were finally output through a global average pooling layer. A multiple instance learning (MIL) framework was integrated into our 2.5D deep learning model, enabling flexible adaptation to tumor lesions with variable slice counts without the need for preprocessing steps such as slice padding or truncation. Specifically, for lesions with fewer than 5 slices, the model directly performs feature extraction and aggregation using the available slices, thereby preserving the original imaging information of small lesions to the greatest extent. For lesions with 5 or more slices, 5 consecutive slices covering the tumor core region were preferentially selected as input; this approach maximizes the retention of tumor spatial heterogeneity and ensures the stability of model performance.

### 2.5 Feature dimensionality reduction, selection, and diagnostic model construction

The extracted radiomic features and 2.5D deep learning features were initially standardized using the z-score to ensure consistency across the entire dataset. The dataset was randomly partitioned into a training set and validation set using a ratio of 7:3. Stratified random sampling was employed with tumor mutation status as the stratification criterion to ensure that the distribution of mutation rates was consistent in the training and validation cohorts. This was followed by t-tests to assess the statistical significance of each feature. Only features with a P-value < 0.05 were retained. To mitigate multicollinearity, Pearson’s correlation coefficients were used to evaluate the correlation between each pair of features, and one feature from each pair with a correlation coefficient > 0.9 was excluded. The least absolute shrinkage and selection operator (LASSO) was applied for dimensionality reduction and feature selection of the extracted DTL and radiomic features. Diagnostic models were constructed using six classifiers: support vector machine, K-nearest neighbor, RandomForest, ExtraTrees, XGBoost, and LightGBM. Sample size was calculated for an expected area under the receiver operating characteristic curve (AUC) value of 0.85 (**Supplementary File 1**). The performance of different diagnostic models was evaluated using the AUC. Decision curve analysis (DCA) was used to assess the clinical net benefit of the models. Calibration curves were plotted to evaluate the goodness-of-fit of the models, and the Hosmer–Lemeshow test was adopted to verify the consistency of the calibration curves.

### 2.6 Statistical analysis

Statistical analyses were performed using Python 3.12, SPSS 26.0, and R 4.2.1 software. The Kolmogorov–Smirnov test was used to assess the normality of continuous data. Normally distributed data were expressed as mean ± standard deviation, while skewed data were presented as quartiles. Categorical data were compared between groups using Pearson’s χ2 test, and continuous data were compared using an independent samples t-test (for normally distributed data) or Mann–Whitney U test (for skewed distributed data).

## 3 Results

### 3.1 Comparison of clinical data

There was no significant difference between the KRAS mutant and wild-type groups in terms of patient age, sex, or tumor markers (carcinoembryonic antigen, carbohydrate antigen 199, or carbohydrate antigen 125) (all P > 0.05) (**Table 1**).

**Table 1:** Comparison of clinical characteristics between KRAS mutant and wild-type rectal cancer patients.

Factor	KRAS mutant (n=43)	KRAS wild-type (n=95)	Statistic	P -value
Age, y(M ± SD)	65.49 ± 11.24	64.18 ± 11.21	0.635^t^	0.527
Gender, n(Male/Female)	33/10	60/35	2.468^a^	0.115
CEAelevation, n(yes/no)	22/21	34/61	2.901^a^	0.089
CA199 elevation, n(yes/no)	14/29	18/77	3.079^a^	0.079
CA125 elevation, n(yes/no)	8/35	16/79	0.064^a^	0.800

^t^t-value; ^a^Chi-square test.

### 3.2 Feature selection and model construction

**Figure 2:**
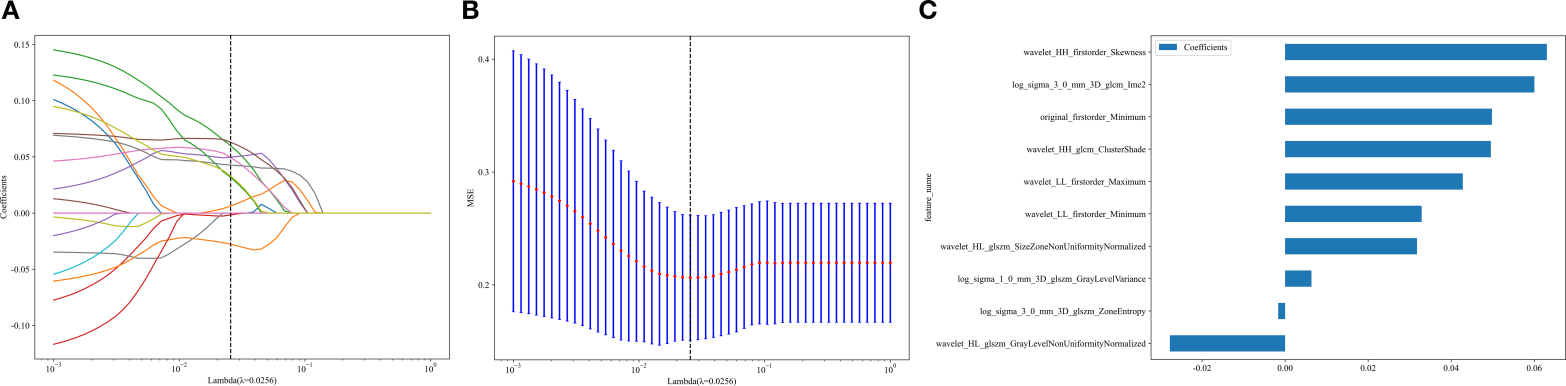
Feature dimensionality reduction and selection for radiomic models (**A, B**: Schematic diagrams of feature selection and dimensionality reduction; **C**: Selected features and their weights after dimensionality reduction and selection).

**Figure 3:**
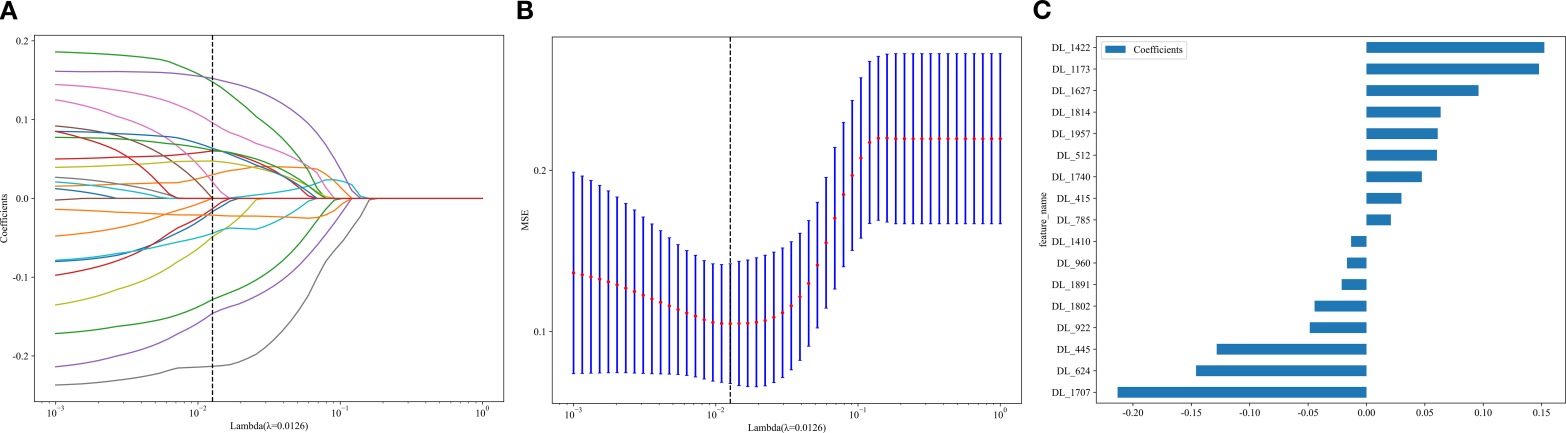
Feature dimensionality reduction and selection for deep transfer learning models (**A, B**: Schematic diagrams of feature selection and dimensionality reduction; **C**: Selected features and their weights after dimensionality reduction and selection).

A total of 960 radiomic features and 2048 DTL features were extracted from the arterial-phase images of each patient. After excluding features with intraclass correlation coefficients < 0.8, followed by dimensionality reduction and selection, 10 radiomic features were used for radiomic model construction and 17 DTL features were used for deep learning model construction (**Figures 2**, **3**).

**Figure 4:**
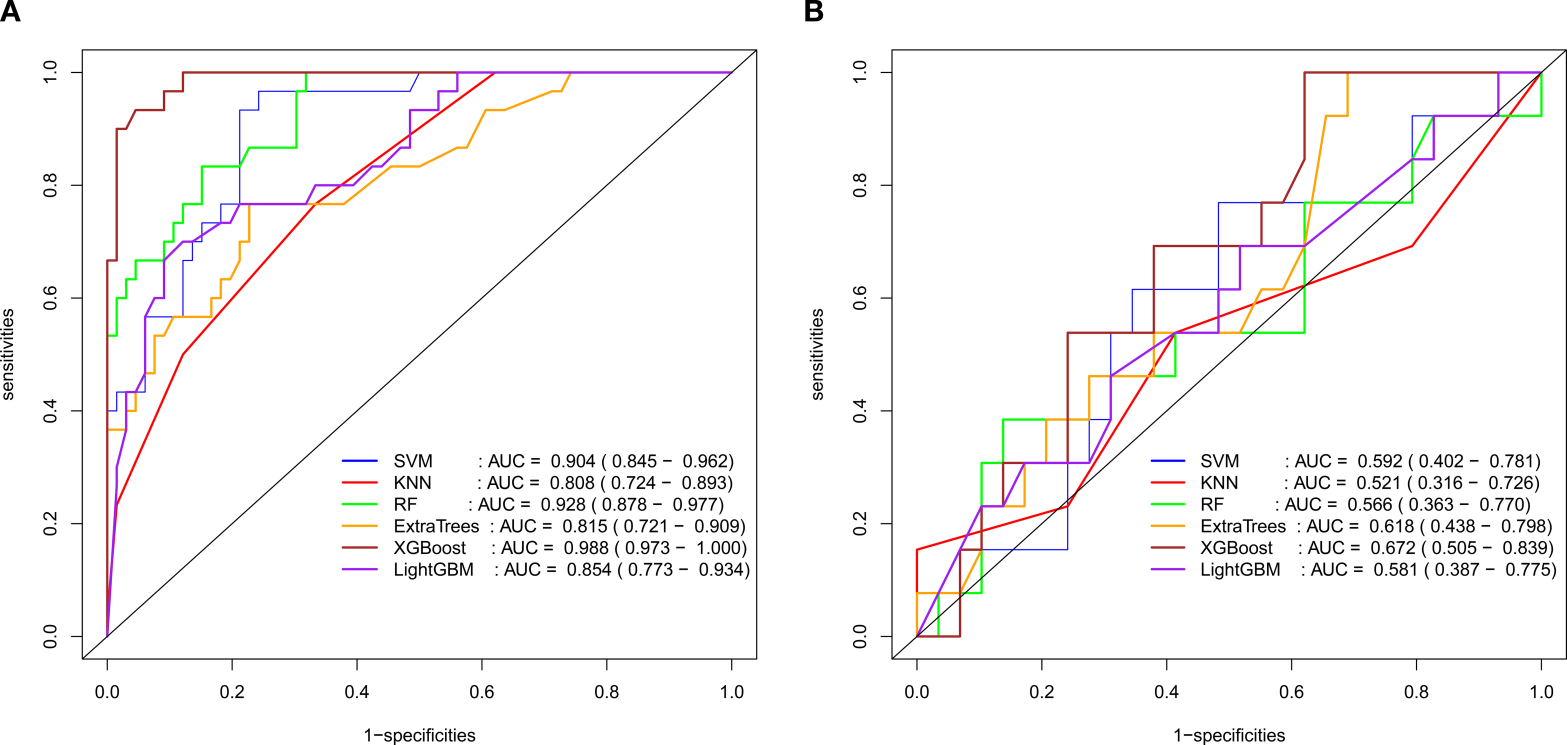
ROC curve results of radiomic diagnostic models (**A**: Training set; **B**: Validation set).

**Figure 5:**
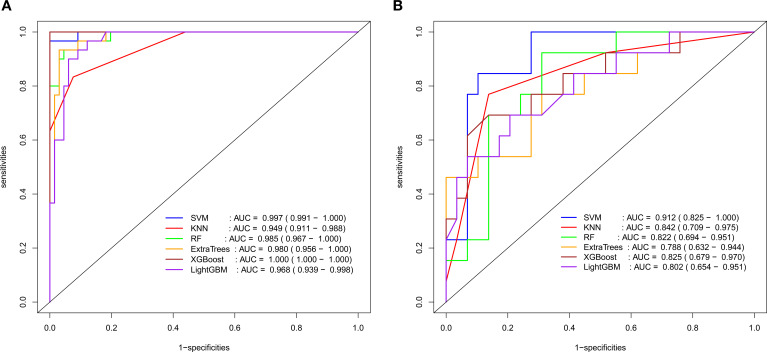
ROC curve results of 2.5D deep learning diagnostic models (**A**: Training set; **B**: Validation set).

Radiomic and 2.5D deep learning models were established based on the selected radiomic and DTL features, respectively. The AUCs of the radiomic models ranged from 0.808–0.988 in the training set and 0.521–0.672 in the validation set, and the AUCs of the 2.5D deep learning models ranged from 0.950–1.000 in the training set and 0.788–0.913 in the validation set. The 2.5D deep learning models exhibited excellent classification performance for identifying KRAS gene mutations in patients with RC, outperforming traditional radiomic models. In the validation set, the optimal classifier for the radiomic models was the XGBoost model (AUC = 0.672), while the best classifier for the deep learning models was the SVM model (AUC = 0.913) (**Tables 2**, **3**; **Figures 4**, **5**).

**Table 2:** Diagnostic performance of radiomic models.

Model	Cohort	AUC (95% CI)	Accuracy	Sensitivity	Specificity
SVM	Training	0.904(0.845-0.963)	0.823	0.967	0.758
	Validation	0.592(0.402-0.781)	0.595	0.769	0.517
KNN	Training	0.808(0.724-0.893)	0.698	0.767	0.667
	Validation	0.521(0.316-0.727)	0.738	0.154	1.000
RF	Training	0.928(0.878-0.977)	0.844	0.833	0.848
	Validation	0.566(0.363-0.770)	0.714	0.385	0.862
ExtraTrees	Training	0.815(0.721-0.909)	0.771	0.767	0.773
	Validation	0.618(0.438-0.798)	0.524	1.000	0.310
XGBoost	Training	0.988(0.973-1.000)	0.948	0.933	0.955
	Validation	0.672(0.506-0.839)	0.571	1.000	0.379
LightGBM	Training	0.854(0.774-0.934)	0.823	0.700	0.879
	Validation	0.581(0.387-0.775)	0.548	0.692	0.483

AUC, the area under the curve; 95% CI, 95% confidence interval; SVM, support vector machines; KNN, K Nearest Neighbors; RF, Random Forest; ExtraTrees, Extreme Gradient Boosting; XGBoost, Extreme Gradient Boosting; LightGBM, Light Gradient Boosting Machine.

**Table 3:** Diagnostic performance of deep learning models.

Model	Cohort	AUC (95% CI)	Accuracy	Sensitivity	Specificity
SVM	Training	0.997(0.991-1.000)	0.990	0.967	1.000
	Validation	0.913(0.825-1.000)	0.881	0.846	0.897
KNN	Training	0.950(0.911-0.988)	0.896	0.833	0.924
	Validation	0.842(0.709-0.975)	0.833	0.769	0.862
RF	Training	0.985(0.967-1.000)	0.948	0.933	0.955
	Validation	0.822(0.694-0.951)	0.762	0.923	0.690
ExtraTrees	Training	0.980(0.956-1.000)	0.958	0.933	0.970
	Validation	0.788(0.632-0.944)	0.833	0.462	1.000
XGBoost	Training	1.000(1.000-1.000)	1.000	1.000	1.000
	Validation	0.825(0.679-0.971)	0.810	0.692	0.862
LightGBM	Training	0.968(0.939-0.998)	0.906	0.967	0.879
	Validation	0.802(0.654-0.951)	0.762	0.692	0.793

AUC, the area under the curve; 95% CI, 95% confidence interval; SVM, support vector machines; KNN, K Nearest Neighbors; RF, Random Forest; ExtraTrees, Extreme Gradient Boosting; XGBoost, Extreme Gradient Boosting; LightGBM, Light Gradient Boosting Machine.

**Figure 6:**
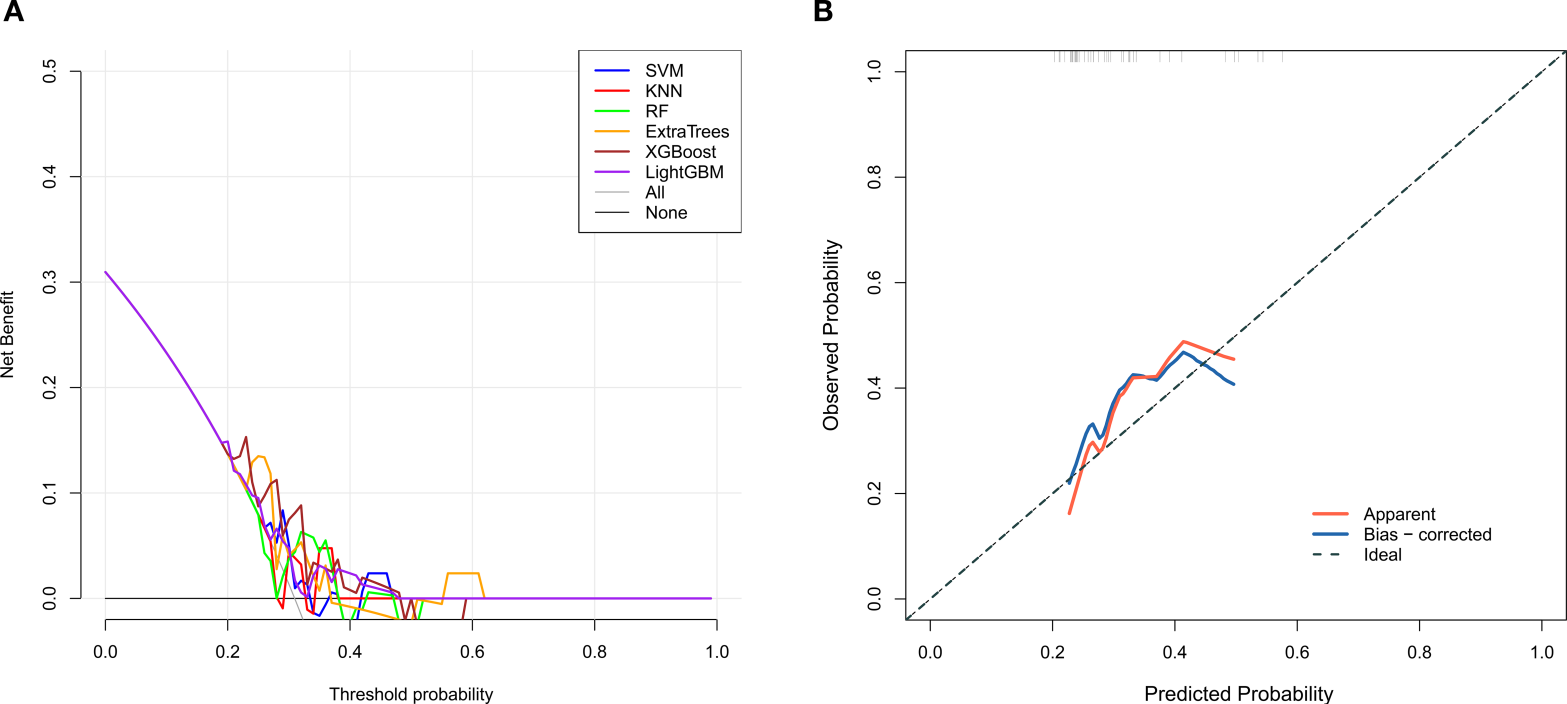
Decision curve analysis (DCA) curves of different radiomics models in the validation set **(A)**; Calibration curve of the radiomics-based XGBoost model in the validation set **(B)**. DCA(Decision curve analysis); Apparent (fitting line); Bias-corrected (deviation correction curve); Ideal (reference line).

**Figure 7:**
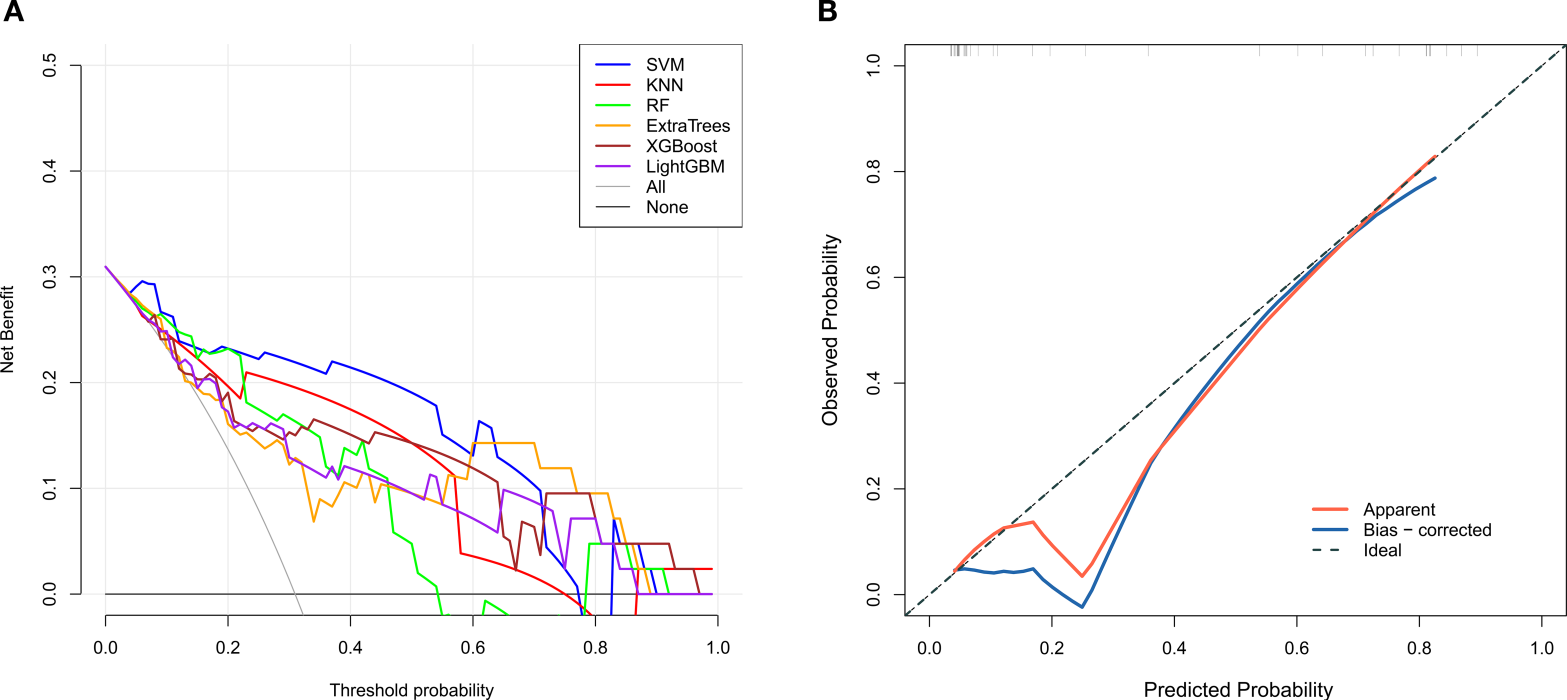
Decision curve analysis (DCA) curves of different 2.5D deep learning models in the validation set **(A)**; Calibration curve of the 2.5D deep learning-based XGBoost model in the validation set **(B)**. DCA(Decision curve analysis); Apparent (fitting line); Bias-corrected (deviation correction curve); Ideal (reference line).

For the radiomic models, the goodness-of-fit of the XGBoost model was assessed using the Hosmer–Lemeshow test, which yielded non-significant statistical results (training set: χ² = 0.467, P = 0.977; validation set: χ² = 4.925, P = 0.295). These findings indicate that the model did not deviate from perfect calibration, and the calibration curves demonstrated favorable calibration performance. DCA revealed that the clinical net benefits of all models were generally comparable in the validation set (**Figure 6**). For the deep learning models, the Hosmer–Lemeshow test was performed to evaluate the goodness-of-fit of the SVM model, with non-significant results obtained (training set: χ² = 1.942, P = 0.747; validation set: χ² = 3.349, P = 0.501). This suggests that the SVM model was perfectly calibrated. The calibration curves further confirmed its satisfactory calibration performance. DCA further showed that the SVM model achieved superior clinical net benefit compared with other models in the validation set (**Figure 7**).

## 4 Discussion

This study explored the construction of diagnostic models based on CT radiomic features and 2.5D deep learning features to distinguish between KRAS mutant and wild-type RC. Six machine learning models were constructed based on radiomic and 2.5D deep learning features. The results showed that the diagnostic performances of the 2.5D deep learning models in the validation set were significantly superior to those of traditional radiomic models (optimal classifier: SVM model with AUC = 0.913), thus providing a novel approach for the preoperative, non-invasive assessment of KRAS mutations in patients with RC.

The KRAS gene is the most commonly mutated oncogene in human cancers, with > 50% of colorectal cancer cases harboring KRAS mutations (11). KRAS mutations can lead to continuous activation of the Ras/Raf/mitogen-activated protein kinase signaling pathway, promoting tumor cell proliferation and rendering anti-epidermal growth factor receptor monoclonal antibody therapy ineffective (12). The early identification of KRAS mutations is therefore crucial to support the personalized treatment of patients with RC. However, traditional post-biopsy genetic testing takes time, and some patients cannot tolerate the trauma of biopsy. There is thus an urgent need for non-invasive and efficient methods for assessing KRAS mutations in patients with RC. Li et al. (13) achieved excellent predictive results for perineural invasion and KRAS mutation in colon cancer using machine learning methods based on preoperative portal venous-phase CT images. Both these studies, however, adopted traditional radiomic methods, which may lack high-dimensional features, thereby affecting model accuracy. In addition, the delineation of 3D tumor regions significantly increases the workload of researchers. In this study, we used 2.5D deep learning technology to construct and validate a model using the maximum tumor slice and two adjacent slices above and below (a total of 5 slices). The resulting 2.5D deep learning model outperformed the traditional 3D radiomic model, in terms of improving the diagnostic performance and reducing the workload of researchers for delineating tumor lesions. Zhao et al. (14) constructed a habitat radiomic model based on 18F-fluorodeoxyglucose-positron emission tomography images of 62 patients to identify KRAS/NRAS/BRAF mutations in patients with RC. The resulting AUC values for the training and validation cohorts were 0.759 and 0.701, respectively. Furthermore, the SHapley Additive exPlanations method indicated that radiomic features derived from the tumor microenvironment had the greatest impact on model prediction. Compared with functional molecular imaging techniques such as 18F-fluorodeoxyglucose-positron emission tomography, CT offers fast imaging, high resolution, and cost-effectiveness.

Magnetic resonance imaging (MRI) can obtain excellent soft tissue resolution and thus performs an important role in evaluating the local infiltration range and lymph node metastasis of RC (15, 16).Cui et al. (17) used radiomic methods based on T2-weighted images and applied three classification methods (logistic regression, decision tree, and support vector machine) to identify KRAS mutations in RC, achieving an AUC of 0.714 in the external validation set. Zhang et al. (18) extracted T2-weighted magnetic resonance imaging radiomic features from 83 patients with RC to predict KRAS mutant and wild-type phenotypes, with a C-index of 0.703 in the validation set, which was lower than that in the present study. In a prospective study, Yuan et al. (19) enrolled 73 patients with RC and collected MRI scans using the intravoxel incoherent motion-diffusion kurtosis imaging sequence. A comparison of the imaging parameters of the KRAS mutant group (apparent diffusion coefficient, true diffusion coefficient, diffusion kurtosis, perfusion fraction, and pseudo-diffusion coefficient) with those of the wild-type group demonstrated that the apparent diffusion coefficient, true diffusion coefficient, and diffusion kurtosis values in the KRAS mutant group were statistically significantly different from those in the wild-type group (P < 0.05). Among these parameters, diffusion kurtosis exhibited the optimal diagnostic performance, with an AUC of 0.779, suggesting that this parameter has a high importance for distinguishing the KRAS gene status. In this study, a 2.5D deep learning model constructed based on CT images showed superior performance in predicting the KRAS gene status of RC patients, and its diagnostic AUC was significantly higher than those reported in the MRI-related studies mentioned above. In addition to its superior diagnostic efficacy, CT examination has distinct advantages in clinical application. On the one hand, CT examination has irreplaceable clinical value for patients with contraindications to MRI scanning. On the other hand, in primary and remote areas with relatively limited medical resources, CT equipment has lower maintenance costs and its examination procedures are convenient and efficient. Thus, it is more suitable for widespread deployment and popularization. The establishment of the model developed in this study is expected to meet the clinical demand for non-invasive assessment of RC gene status in primary medical institutions.

DTL features based on the ResNet pre-trained model can effectively capture macro-imaging features such as tumor edge characteristics and overall spatial distribution, thereby accurately identifying imaging differences under different gene mutation states (20). In addition, this method achieves efficient learning of high-level semantic features in medical images by transferring general features from the field of natural images, which can reduce the risk of overfitting in small-sample medical-data scenarios. This may be the key reason why the deep learning model in this study maintained a stable predictive performance in the validation set. Gan et al. (21) predicted the *KRAS* mutation status of RC patients using transrectal ultrasound images, and their findings indicated that the diagnostic performance of the deep learning model constructed based on the ResNet50 deep learning network outperformed that of the conventional radiomic model. Yang et al. (22) successfully predicted lymph node metastasis in RC patients by establishing a deep learning model, whereas Sun et al. (23) applied a multiparametric MRI-based deep learning model to identify synchronous liver metastases in RC patients. Collectively, these studies have demonstrated that deep learning models have substantial clinical utility for predicting critical clinical indicators in patients with RC, including prognosis and gene mutation status. Currently, relevant research on 2.5D deep learning models for RC patients remains relatively limited. Nevertheless, this technology has exhibited excellent predictive performance in the diagnosis and prognostic evaluation of other clinical lesions, thereby providing precedence for its application in the field of RC. For example, Cen et al. (24) successfully predicted the early recurrence of hepatocellular carcinoma by constructing a 2.5D deep learning model. Li et al. (25) constructed a predictive model for diffuse gliomas using preoperative multiparametric magnetic resonance imaging. They adopted the ResNet18 deep learning network and selected images of the maximum tumor diameter and two adjacent slices above and below to construct a 2.5D deep learning framework. They showed that the predictive performance of this 2.5D model was significantly superior to that of traditional radiomic models, with an AUC of 0.85–0.89 in the validation set. Huang et al. (26) also confirmed the advantages of 2.5D deep learning. Their constructed model successfully predicted occult lymph node metastasis in lung adenocarcinoma, with a performance superior to traditional radiomic models, consistent with the conclusions of the current study. The predictive models constructed based on six machine learning models in this study all showed better performance than the radiomic models in the validation set. Further related studies comparing the predictive performances of 2.5D and 3D deep learning models confirmed the diagnostic advantages of 2.5D models (27, 28). First, by integrating 2D slices of multiple key layers, 2.5D models effectively retain core spatial correlation information for the ROI without the need to construct a complete 3D structure. Second, compared with 3D models, 2.5D models require less training data, enabling stable model training even with a small number of samples. They also require fewer computational resources, and data delineation only needs to focus on the key layers, thus greatly reducing the time and labor costs of data annotation and preprocessing. Third, compared with traditional 2D models, 2.5D models can capture complex anatomical structure details and disease-specific features in images more comprehensively and accurately through multi-slice information fusion, ultimately improving prediction accuracy while ensuring the efficiency and practicality of clinical application.

The results demonstrated that of the radiomics models, the XGBoost model achieved the optimal diagnostic performance, with an AUC of 0.988 in the training set; however, the AUC decreased to 0.672 in the validation set, indicating poor model stability and an elevated risk of overfitting. This phenomenon could be attributed to the relatively small sample size and excessively high dimensionality of the extracted features in the present study. When the feature dimensionality is far greater than the sample size, the data tend to exhibit a sparse distribution in the high-dimensional space, leading to the so-called “curse of dimensionality” (29). In contrast, the SVM prediction model constructed based on 2.5D deep learning yielded an AUC of 0.997 in the training set and 0.913 in the validation set, with an extremely small discrepancy in performance between the two sets and no evidence of model overfitting.

This study had several limitations. First, it was a retrospective study and thus inevitably subject to selection bias. Second, it was a single-center, small-sample study, and further validation is needed using large-sample, multi-center data. Third, image delineation was performed manually and was thus inevitably affected by the subjectivity of the delineator. Further studies should apply more advanced artificial intelligence algorithms to improve the efficiency and accuracy of segmentation. Finally, this study used arterial-phase enhanced data for model construction, and future research should verify the ability of multi-parametric imaging data to improve model diagnostic performance.

## 5 Conclusions

2.5D deep learning models can effectively distinguish between KRAS mutant and wild-type RC, with better diagnostic performance than traditional radiomic models, thus providing a new non-invasive method and insights for the preoperative assessment of patients with RC.

## Data Availability

The original contributions presented in the study are included in the article/**Supplementary Material**. Further inquiries can be directed to the corresponding authors.
