# Supplementary material for: Identification of KRAS mutation in rectal cancer based on a 2.5D deep learning model
Source: Front Oncol. 2026 Feb 25;16:1763859. doi: 10.3389/fonc.2026.1763859 (PMC12975492; doi:10.3389/fonc.2026.1763859)
Supplement: Supplementary file 1 [file DataSheet1.pdf]

1.Sample size: area under ROC curve

Options

Type I error (Alpha, Significance)	0.05
Type II error (Beta, 1-Power)	0.10

Data

Area under ROC curve	0.85
Null Hypothesis value	0.50
Ratio of sample sizes in negative / positive groups	2

Result

Number of positive cases required:	9
Number of negative cases required:	18
Total sample size (both groups together)	27

Table

		Type I Error - Alpha			
		0.20	0.10	0.05	0.01
Type II Error - Beta	0.20	4 + 8	6 + 12	7 + 14	11 + 22
	0.10	6 + 12	7 + 14	**9 + 18**	13 + 26
	0.05	7 + 14	9 + 18	11 + 22	15 + 30
	0.01	10 + 20	12 + 24	14 + 28	19 + 38

Sunday, January 25, 2026 09:44 - MedCalc® version 23.4.0

2. Adjustment for Multiple Comparisons (6 Classifiers)

To account for the inflated Type I error rate when comparing 6 classifiers, we applied an empirical expansion factor:

$$\text{Expansion factor} = 1 + (k-1) \times 0.1 = 1 + (6-1) \times 0.1 = 1.5$$

where K is the number of classifiers.

After adjustment:

*Total sample size for 6 classifiers: 27*1.5=41*

*Positive cases: 9*1.5≈14*

*Negative cases:18*1.5≈27*
